# Integrating Clinical, Brain Imaging, and Biomarker Big Data to Elucidate Links Between Depression and Subsequent Memory Disorders – A Protocol Description

**DOI:** 10.1192/j.eurpsy.2026.11610

**Published:** 2026-07-31

**Authors:** N. M. Scheinin, S. Kline, M. Mäkinen, J. J. Tuulari, H. Merisaari

**Affiliations:** 1 Department of Psychiatry, University of Turku, Turku; 2 Department of Psychiatry, Satakunta Wellbeing Services County, Pori; 3 Turku Brain and Mind Center, University of Turku; 4 Department of Radiology, University of Turku and Turku University Hospital, Turku, Finland

## Abstract

**Introduction:**

Depression has been linked to increased risk of memory disorders, but whether it exerts a causal effect remains uncertain. Even large-scale Mendelian randomization studies have not been able to reconcile whether the association is causal. The “cognitive scarring” hypothesis posits that depressive episodes may lead to structural or functional brain changes that reduce cognitive reserve and predispose to subsequent memory disorders.

**Objectives:**

The project aims to elucidate whether prior major depressive disorder (MDD) is associated with specific brain change patterns, detectable by structural MRI, that could explain reduced cognitive reserve and increased vulnerability to memory decline. The aim is to study these potential neural correlates of MDD in a cohort containing patients with mild cognitive impairment and Alzheimer’s disease, as well as healthy age- and sex-matched controls.

**Methods:**

We are establishing a large-scale biobank-based study integrating structural brain MRI, clinical, and extensive genetic and biomarker data to examine these potential pathways. We currently have clinical MRI scans from 1600, 1600, and 1500 individuals, for the modalities T1, T2, and FLAIR. The dataset is complemented by extensive registry-linked information, including diagnoses, genetic information, symptom scale results, and blood and cerebrospinal fluid biomarkers. The Finnish biobank framework enables comprehensive data linkage across registries under uniform ethical and legal standards. A dedicated AI-based harmonization pipeline is being developed to correct for scanner model and imaging protocol heterogeneity in the clinically acquired MRIs, allowing robust volumetric and morphometric analyses across modalities and sites. A sub-cohort with follow-up MRIs will be formed to enable complementary longitudinal analyses.

**Results:**

The project can reveal, with high statistical power, whether individuals with prior MDD show distinct brain signatures identified from structural MRIs, compared to those without such a history, and whether these differences mediate risk for cognitive decline. Further, the roles of genetics and several biomarkers as potential mediators or modifiers of the associations will be elucidated.

**Conclusions:**

By combining neuroimaging, clinical, genetic, and biomarker data within a unique national biobank infrastructure, this study provides an unprecedented opportunity to test the neurobiological plausibility of depression as a causal risk factor for memory disorders. The hybrid design with cross-sectional and longitudinal data is optimal for a) finding novel associations, b) understanding linking mechanisms, and c) inferring causality in the relationships. The findings will aid in developing targeted prevention strategies for memory disorders.

**Disclosure of Interest:**

None Declared

